# Best Practices for Successfully Writing and Publishing a Genome Announcement in *Microbiology Resource Announcements*

**DOI:** 10.1128/MRA.00763-20

**Published:** 2020-09-03

**Authors:** Julie C. Dunning Hotopp, David A. Baltrus, Vincent M. Bruno, John J. Dennehy, Steven R. Gill, Julia A. Maresca, Jelle Matthijnssens, Irene L. G. Newton, Catherine Putonti, David A. Rasko, Antonis Rokas, Simon Roux, Jason E. Stajich, Kenneth M. Stedman, Frank J. Stewart, J. Cameron Thrash

**Affiliations:** a Institute for Genome Sciences, University of Maryland School of Medicine, Baltimore, Maryland, USA; b Department of Microbiology and Immunology, University of Maryland School of Medicine, Baltimore, Maryland, USA; c Greenebaum Cancer Center, University of Maryland School of Medicine, Baltimore, Maryland, USA; d School of Plant Sciences, University of Arizona, Tucson, Arizona, USA; e School of Animal and Comparative Biomedical Sciences, University of Arizona, Tucson, Arizona, USA; f Biology Department, Queens College, City University of New York, Flushing, New York, USA; g The Graduate Center, City University of New York, New York, New York, USA; h Department of Microbiology and Immunology, University of Rochester School of Medicine and Dentistry, Rochester, New York, USA; i Department of Civil and Environmental Engineering, University of Delaware, Newark, Delaware, USA; j University of Leuven, Department of Microbiology, Immunology, and Transplantation, Rega Institute, Leuven, Belgium; k Department of Biology, Indiana University, Bloomington, Indiana, USA; l Bioinformatics Program, Loyola University Chicago, Chicago, Illinois, USA; m Department of Biology, Loyola University Chicago, Chicago, Illinois, USA; n Department of Microbiology and Immunology, Stritch School of Medicine, Loyola University Chicago, Maywood, Illinois, USA; o Department of Biological Sciences, Vanderbilt University, Nashville, Tennessee, USA; p DOE Joint Genome Institute, Lawrence Berkeley National Laboratory, Berkeley, California, USA; q Department of Microbiology and Plant Pathology, University of California, Riverside, Riverside, California, USA; r Center for Life in Extreme Environments, Portland State University, Portland, Oregon, USA; s Biology Department, Portland State University, Portland, Oregon, USA; t Department of Microbiology and Immunology, Montana State University, Bozeman, Montana, USA; u School of Biological Sciences, Georgia Institute of Technology, Atlanta, Georgia, USA; v Department of Biological Sciences, University of Southern California, Los Angeles, California, USA

## Abstract

*Microbiology Resource Announcements* (MRA) provides peer-reviewed announcements of scientific resources for the microbial research community. We describe the best practices for writing an announcement that ensures that these publications are truly useful resources. Adhering to these best practices can lead to successful publication without the need for extensive revisions.

## EDITORIAL

*Microbiology Resource Announcements* (MRA) provides peer-reviewed announcements of scientific resources for the microbial research community. Such resources include genomes, transcriptomes, amplicon sequence data sets, other sequence collections, culture collections, mutant libraries, and software (Table 1). The MRA editors have established minimum requirements needed to ensure that the laboratory and analytical methods can be replicated by any other research group, making these publications truly useful resources for the community. These minimum requirements are described in checklists for each resource type (https://mra.asm.org/sites/default/files/additional-assets/thumbs/MRA_Author_Checklist.pdf). Adhering to the relevant checklist streamlines the review process for both authors and reviewers. In this guide, we take the most common resource, the genome announcement, and provide detailed recommendations for the three main sections of a genome announcement. Use of these best practices can lead to successful publication without the need for extensive revisions.

**TABLE 1:** Example published resource announcements in MRA

Resource announcement type^*a*^	Example(s)
Complete circular bacterial genome	Complete genome sequence of *Pseudomonas coronafaciens* pv. oryzae 1_6 (11); Complete genome sequence of *Luteibacter pinisoli* MAH-14 (12)
Draft bacterial genome with Illumina data only	Draft genome sequence of *Lactobacillus jensenii* strain UMB7766, isolated from the female bladder (13)
Bacterial genome with PacBio data only	Draft genome sequence of a fish pathogen, *Edwardsiella piscicida* isolate CK41 (14)
Bacterial genome with ONT data only	Complete genome sequence of halophilic deep-sea bacterium *Halomonas axialensis* strain Althf1 (15)
Obligate host-associate bacterial genome	Complete genome sequence of *w*Ana, the *Wolbachia* endosymbiont of *Drosophila ananassae* (2)
Many genomes from a large bacterial culture collection	Eleven high-quality reference genome sequences and 360 draft assemblies of Shiga toxin-producing *Escherichia coli* isolates from human, food, animal, and environmental sources in Canada (16)
Phage double-stranded DNA genome	Complete genome sequence of *Escherichia coli* phage Paul (17); Complete genome sequence of *Escherichia coli* siphophage Snoke (18)
Linear viral RNA genome	Complete genome sequence of an American isolate of pepino mosaic virus (19)
Small circular viral single-stranded DNA genome	Genome sequences of three cruciviruses found in the Willamette Valley (Oregon) (20)
Draft microbial eukaryotic genome with Illumina data only	Draft genome sequence of the griseofulvin-producing fungus *Xylaria flabelliformis* strain G536 (21)
Draft microbial eukaryotic genome with PacBio data	Genome sequence of the extremely acidophilic fungus *Acidomyces richmondensis* FRIK2901 (22); Genome sequence of a California isolate of *Fusarium oxysporum* f. sp. *lycopersici* race 3, a fungus causing wilt disease on tomato (23)
Complete microbial eukaryotic genome	Complete genome sequences for two *Talaromyces marneffei* clinical isolates from northern and southern Vietnam (24)
Nearly complete microbial eukaryotic genome	Nearly complete genome sequence of *Brugia malayi* strain FR3 (25)
16S amplicon study	16S rRNA amplicon sequencing of sediment bacterial communities in an oyster farm in Rhode Island (26)
Transcriptome	Multispecies transcriptomics data set of *Brugia malayi*, its *Wolbachia* endosymbiont *w*Bm, and *Aedes aegypti* across the *B. malayi* life cycle (27)
Culture collections/mutant libraries	Gateway entry vector library of *Wolbachia pipientis* candidate effectors from strain *w*Mel (28)
Metagenome	Microbiota of the Hickey Run Tributary of the Anacostia River (29); Metagenomic assembly and prokaryotic metagenome-assembled genome sequences from the northern Gulf of Mexico “dead zone” (30)
Metatranscriptome	Metatranscriptomic sequencing of a cyanobacterial soil-surface consortium with and without a diverse underlying soil microbiome (31)
Software	TwinBLAST: when two is better than one (32)
Organelle genome	Complete chloroplast genome sequence of a white spruce (*Picea glauca*, genotype WS77111) from eastern Canada (33); Mitochondrial genome sequences of *Diorhabda carinata* and *Diorhabda carinulata*, two beetle species introduced to North America for biological control (34)
Proteome	Proteome of a *Moraxella catarrhalis* strain under iron-restricted conditions (35)

a PacBio, Pacific Biosciences; ONT, Oxford Nanopore Technologies.

## INTRODUCTION AND RATIONALE FOR SEQUENCING

The first section of a genome announcement should provide a brief introduction that focuses on the rationale for or significance of sequencing. This introduction should reference appropriate literature but, due to space constraints, should not be an exhaustive review. A greater emphasis should be put on introducing the characteristics of the isolate(s) and providing a description of the provenance of the organism(s) in a manner that supports using the genome as a resource. The best practice is to comply with the Genomic Standards Consortium Minimum Information about any (x) Sequence (MIxS) checklist (1) (https://gensc.org/mixs), ensuring consistency with the same information available in the biosample accession record or equivalent. Type strains can be noted if the strain is listed as such in a type strain repository, such as the American Type Culture Collection (ATCC), or on a reference website, such as https://bacterio.net. We do not allow claims of priority (e.g., first or novel); all genomes are new in their own unique ways without such claims.

Although an MRA genome announcement requires a taxonomic designation for the organism at the genus level, journal policy does not allow formal descriptions of sequenced organisms as new species, proposals for new taxonomy, or proposals for taxonomic reorganization. Such designations typically require much greater evidentiary support than can be reasonably included in a genome announcement. A formal taxonomic description should be directed to the *International Journal of Systematic and Evolutionary Microbiology* or *Archives of Virology*. Therefore, for genome announcements, the taxonomic nomenclature should have been described previously and should be consistent with established nomenclature rules (e.g., listed by https://bacterio.net). If authors would like to submit a resource describing the genome of an organism that does not have a species designation, the authors can designate the organism as a member of an existing genus (e.g., *Wolbachia* sp. strain *w*Ana [2]) and note the organism’s similarity to, or difference from, its closest relative(s) using appropriate techniques and analyses that are fully described.

The genome announcement should contain a description of how the isolate was acquired. This may include an accession number for a public culture collection, like the ATCC, BEI Resources, the ARS Culture Collection (NRRL), the German Collection of Microorganisms and Cell Cultures (DSMZ), or the Japanese Collection of Microorganisms. Adding information about how the strain was acquired and maintained can be helpful to others trying to interpret the sequencing results. It can be important to understand whether the specimen was acquired directly from a culture collection or from another scientist, along with a relative time frame and method for storing and/or passaging. If a new isolate is described, the genome announcement should include a description of when, where, and how the organism was isolated. A brief description can be followed by a citation to a peer-reviewed manuscript for the full isolation procedures. For new environmental isolates, the latitude and longitude or GPS coordinates of the sample site should be included. For clinical isolates, the best practice is to adhere to the metadata standards for human pathogen/vector genomic sequences (3). Authors of manuscripts describing research involving human or animal subjects must include a statement documenting the approval number and name of their institutional review board (IRB) or institutional animal care and use committee (IACUC). In all cases, the authors should make the sequenced isolate available to the community upon request; the best practice is to deposit the isolate in a culture repository (4).

While MRA does not allow for the inclusion of extensive experimental results, the rationale for sequencing may include a figure or table illustrating a specific trait, such as a strain’s ability to produce fungicides or an antibiotic resistance profile. Those methods must be fully described in the main text or the figure legend with sufficient detail and/or references to be reproduced. If new antibiotic resistance profiles are included, the Clinical and Laboratory Standards Institute (CLSI) standards and methods used to determine antibiotic resistance should be fully described. Elaborate phenotypic results should not be included, as these often require extensive experimental validation. MRA does not allow references to unpublished results or personal communications.

## SEQUENCING, ASSEMBLY, AND OTHER BIOINFORMATICS METHODS

Typically, the second section of a genome announcement includes a description of the methods and related outcomes. The paragraph must describe the methods for organism cultivation/acquisition, any taxonomic identification, DNA/RNA isolation, sequencing library preparation, and sequence generation, including platform specifications. The goal of this paragraph is to ensure that the sequencing procedure can be fully replicated and to enable full data reuse; therefore, details such as manufacturer, kit identifiers, and/or modifications to published protocols are essential. For Illumina sequencing, the platform should be described, along with read pairing status, the length of the reads, the number of raw reads in total and/or the sequencing depth, and the methods for quality control and trimming, if applicable. For Pacific Biosciences (PacBio) sequencing, the platform should be described, preferably with the chemistry, along with the library construction method, whether and how DNA was sheared, whether and how DNA was size selected, the read *N*_50_, the number of raw reads, and, if applicable, a description of read quality control, error correction, and adapter trimming. For Oxford Nanopore Technologies sequencing, the device and flow cell should be described, along with the library construction methods, the read *N*_50_ and number of raw reads, the base caller, read quality control, error correction, and, if applicable, adapter trimming. For libraries constructed with the ligation method (i.e., not RAD/RAPID libraries), whether and how DNA was sheared and size selected should be described. For capillary sequencing, which is still frequently used to sequence some viral genomes, the primers used to amplify and sequence the genome should be provided, which is often best accomplished in a table. The sequencing instrument should be specified, along with the length distribution of the reads, how the reads overlapped, and the Phred quality score threshold used for read trimming.

After describing the sequencing and preassembly quality control methods, the genome assembly and annotation methods should be fully described. Annotation methods vary widely between taxa, such that it is important to follow the best practices in the field, but a few principles are common across all taxa. All software should be cited and a version number included, even for common software like PGAP (5). Settings or options used to run the software should be provided, and we encourage including a statement such as “default parameters were used, except where otherwise noted,” if appropriate. Custom scripts must be made publicly available, and a link with a permanent DOI should be provided for the scripts (e.g., a GitHub repository with an assigned DOI using the data-archiving tool Zenodo). The annotation described in the genome announcement should be consistent with the data that are publicly available with the genome accession number(s) listed in the data availability section.

## DESCRIBING THE RESULTS OF THE GENOME SEQUENCING

The final section describes the results, including the complete size, GC content, and final sequencing coverage of the genome. For genome announcements that include the genomes of more than one strain or organism, it is helpful to include a table with this information. That table should also include hyperlinked accession numbers for the genome and the raw data. For draft genomes, the announcement should include the relevant statistics for the assembly, including the number of contigs and the contig *N*_50_ value, as well as any method for ordering and orienting contigs. It should be clear what criteria, if any, were used for removal of contigs due to size or contamination screening. For complete linear genomes, this section describes how the ends of the chromosomes/genome were determined to be complete. For circular genomes, the method for identifying the overlap on the contig ends, trimming, and rotating, if applicable, should be specified.

Genome quality assessment using a conserved set of markers can be a useful and important metric for evaluating whether the genome is complete, particularly for large genomes. Assessment with tools like BUSCO (6), CEGMA (7), or CheckM (8) and/or whole-genome reference alignments with tools like MUMmer (9) or PacBio Quiver can be helpful for reporting genome completeness and duplication metrics but may not be universally appropriate. Visualization of assembly graphs can be helpful for assessing completeness using tools like Bandage (10).

Any remaining space is typically dedicated to describing the results in the context of the rationale for sequencing described in the introduction. Authors should avoid claims that imply that a particular gene or operon is functional in a sequenced organism. Functions of genes should be presumed and noted as “putative” or “potential,” unless they have been functionally characterized previously and a reference is provided.

A main figure can include a phylogeny to present the relationship of the new genome resource(s) to other isolates or species. When a phylogeny is included, the methods should be rigorous and fully described in the main text or the figure legend, including the procedures used to select the included sequences, accession numbers for the included sequences, multiple sequence alignment, alignment curation, model selection, tree building with statistical support, and tree visualization.

## DATA AVAILABILITY STATEMENT

All American Society for Microbiology (ASM) journals require the inclusion of a sufficient amount of publicly available data so that others can reproduce analyses and results from the published manuscripts. A list of acceptable databases is available (https://journals.asm.org/content/list-data-repositories). For MRA, the underlying data largely consist of sequencing reads and genome assemblies. Accession numbers hyperlinked directly to raw sequencing reads (e.g., SRX, SRR, or SRP accession numbers), from one of the acceptable repositories (International Nucleotide Sequence Database Collaboration [INSDC] [http://www.insdc.org], e.g., SRA or ENA), should be listed in the data availability section. Although it is acceptable to deposit reads that have been modified (for instance, through quality trimming), we ask that authors consider depositing the least modified reads and specify any types of modifications in the data availability statement. Data sets that include human reads should be placed in an appropriate repository, like dbGaP. For large collections of genomes, the data availability section can refer to a table in which the read accession numbers are provided for each isolate.

## CONCLUSIONS

If these elements are all included and correct and no problems are identified with the rationale or conclusions, a manuscript could be designated by the editors a unicorn—a paper that is accepted on first submission without revisions. Interested in making your submission the next unicorn? Use the checklist and the examples (Table 1) to guide you in constructing a solid genome announcement. And remember, you can have one figure and one table to support your genome announcement.
